# Engagement with a nationally-implemented digital behaviour change intervention: Usage patterns over the 9-month duration of the National Health Service Digital Diabetes Prevention Programme

**DOI:** 10.1016/j.invent.2023.100647

**Published:** 2023-07-12

**Authors:** Rhiannon E. Hawkes, Lisa M. Miles, Ben Ainsworth, Jamie Ross, Rachel Meacock, David P. French

**Affiliations:** a Manchester Centre for Health Psychology, Division of Psychology and Mental Health, University of Manchester, UK; b Department of Psychology, University of Bath, UK; c School of Psychology, University of Southampton, UK; d Centre for Primary Care, Wolfson Institute of Population Health, Barts and The London School of Medicine and Dentistry, Queen Mary University of London, UK; e Health Organisation, Policy and Economics (HOPE) Research Group, Centre for Primary Care and Health Services Research, University of Manchester, UK

**Keywords:** Diabetes prevention, Digital interventions, User engagement, Behaviour change

## Abstract

**Background:**

Digital behaviour change interventions may offer a scalable way to promote weight loss by increasing physical activity and improving diet. However, user engagement is necessary for such benefits to be achieved. There is a dearth of research that assesses engagement with nationally implemented digital programmes offered in routine practice. The National Health Service Digital Diabetes Prevention Programme (NHS-DDPP) is a nine-month digital behaviour change intervention delivered by independent providers for adults in England who are at high risk of developing type 2 diabetes. This study reports engagement with the NHS-DDPP for users enrolled onto the programme over the nine-month duration.

**Methods:**

Anonymous usage data was obtained for a cohort of service users (*n* = 1826) enrolled on the NHS-DDPP with three independent providers, between December 2020 and June 2021. Usage data were obtained for time spent in app, and frequency of use of NHS-DDPP intervention features in the apps including self-monitoring, goal setting, receiving educational content (via articles) and social support (via health coaches and group forums), to allow patterns of usage of these key features to be quantified across the nine-month intervention. Median usage was calculated within nine 30-day engagement periods to allow a longitudinal analysis of the dose of usage for each feature.

**Results:**

App usage declined from a median of 32 min (IQR 191) in month one to 0 min (IQR 14) in month nine. Users self-monitored their behaviours (e.g., physical activity and diet) a median of 117 times (IQR 451) in the apps over the nine-month programme. The open group discussion forums were utilised less regularly (accessed a median of 0 times at all time-points). There was higher engagement with some intervention features (e.g., goal setting) when support from a health coach was linked to those features.

**Conclusions:**

App usage decreased over the nine-month programme, although the rate at which the decrease occurred varied substantially between individuals and providers. Health coach support may promote engagement with specific intervention features. Future research should assess whether engagement with particular features of digital diabetes prevention programmes is associated with outcomes such as reduced bodyweight and HbA1c levels.

## Highlights

• Engagement with a nationally implemented digital programme delivered by providers.
• Self-monitoring of behaviours was used regularly across the 9-month programme.
• Group support features were rarely utilised across providers.
• Health coach support may promote engagement with specific programme features.

## 1 Introduction

Type 2 diabetes mellitus (T2DM) is an international public health issue; global prevalence increased to 422 million in 2014 (World Health Organization, 2016), and 13.6 million people in the United Kingdom (UK) are currently at increased risk of developing T2DM (Diabetes UK, 2020a). However, diabetes prevention trials have shown that progression to T2DM in people at high risk could be slowed or prevented by weight loss via changes in behaviours, notably increased physical activity and improved nutrition (e.g., (World Health Organization, 2016; Diabetes UK, 2020a; Gong et al., 2019; Tuomilehto et al., 2001; Knowler et al., 2002; Kosaka et al., 2005; Ramachandran et al., 2006)).

Following this international evidence, the National Health Service (NHS) in England launched the NHS Diabetes Prevention Programme (NHS-DPP) in 2016; a nine-month face-to-face behavioural intervention for adults in England identified as being at increased risk of developing T2DM (NHS England, 2021). Initial results have been promising; the NHS-DPP has been shown to help people at risk to achieve weight loss (Valabhji et al., 2020; Marsden et al., 2022) and has reduced population incidence of T2DM (McManus et al., 2022). However, targets for programme improvement have been identified which digital technologies could address, such as increasing access and engagement for younger adults and those in employment (Howarth et al., 2020).

Digital interventions are attractive as they have the potential to be rolled out at scale compared to programmes delivered in community settings (Grock et al., 2017). In line with this, NHS England launched a pilot of the Digital Diabetes Prevention Programme (NHS-DDPP) (Murray et al., 2019). Initial results on the outcomes of the NHS-DDPP pilot have shown clinically significant reductions in weight and blood glucose levels (hemoglobin A1c [HbA1c]), thus demonstrating a reduction in T2DM risk in those who took part in the digital pilot programme (Ross et al., 2022). More recently, the digital pilot programme was shown to be as effective as the face-to-face programme in preventing T2DM (Marsden et al., 2023). Subsequently, a digital pathway was introduced to the programme in 2019 (NHS England, 2019), with four independent providers commissioned to deliver the digital service in localities across England. The four NHS-DDPP providers designed their own versions of the digital intervention in line with evidence reviews (Hawkes et al., 2022a), which was offered via modalities including apps, educational platforms and health coach support to help people make changes to their diet and activity behaviours (Miles et al., 2023a; Hawkes et al., 2023).

However, for service users to benefit from these digital behaviour change programmes, they have to engage with features of the intervention (e.g., monitoring behaviours via apps, educational content, receiving support) before they can understand and use the key behaviour change content (Hankonen, 2021). There is good evidence that behaviour change techniques (BCTs, e.g., monitoring behaviours, setting goals) are the ‘active ingredients’ that produce behaviour change in individuals (Michie et al., 2013). Digital interventions provide the opportunity to analyse usage of BCTs and other intervention features through routinely collected usage data, using frameworks (e.g., AMUsED: Analyzing and Measuring Usage and Engagement Data; (Miller et al., 2019)) to identify digital metrics that signify meaningful engagement from target users. Such analyses can highlight effective components that translate to real-world behaviour change in service users that is likely to lead to improved health outcomes; known as ‘effective engagement’ (Yardley et al., 2016).

Randomised controlled trials have assessed user engagement of digital behaviour change interventions to prevent T2DM over six (Block et al., 2015; Harjumaa et al., 2020) and 12 months (Lavikainen et al., 2022; Moin et al., 2018; Painter et al., 2020; Toro-Ramos et al., 2020; Sepah et al., 2017). Crucially, programme engagement was linked with reductions in fasting glucose and HbA1c (Block et al., 2015), increased physical activity (Batten et al., 2022), higher increase in diet quality (Lavikainen et al., 2022) and decreased BMI or weight loss (Lavikainen et al., 2022; Moin et al., 2018; Toro-Ramos et al., 2020; Sepah et al., 2017; Batten et al., 2022). One study analysed usage data from a digital programme designed to support self-management of T2DM, which was integrated into routine care in four Clinical Commissioning Groups in London, UK (Poduval et al., 2018). This study compared usage across demographic characteristics and found no evidence of widening health inequalities in terms of usage (Poduval et al., 2018).

To date, no studies have reported on actual usage and engagement of large-scale nationally implemented programmes. It is widely acknowledged that many interventions are less effective in routine practice than in randomised trials (i.e. “voltage drop” (Chambers et al., 2013)), at least partly because people enrolled in trials tend to be highly motivated and hence may engage more than people who receive interventions as part of routine practice. Thus it is possible that, when rolled out, many people who are referred to the NHS-DDPP may use it only briefly or not at all, thereby limiting its effectiveness. Previous research on the NHS-DDPP has found variation in the number and types of BCTs that are offered via app, educational articles and health coach support (Hawkes et al., 2023). For example, if people do not engage with the articles containing educational content, they could miss key BCTs in the programme. Thus, it is crucial to assess the extent to which users engage with these different modes of delivery and therefore, access and potentially use, these BCTs.

This study aimed to understand engagement with the NHS-DDPP for a cohort of service users enrolled with service providers over the nine-month programme duration. Specific objectives were to: (1) describe duration of engagement on the app, (2) describe overall frequency of use of intervention features, (3) describe patterns across time in engagement with intervention features over the nine-month duration, and (4) compare any differences in engagement with intervention features between three of the NHS-DDPP providers over time.

## 2 Methods

### 2.1 Study design

This study analysed routinely collected usage data from a cohort of users on the nationally implemented English NHS-DDPP who started the programme between December 2020 and June 2021. Usage data was collected for the nine-month duration of the intervention. Data was requested from all four independent service providers delivering the NHS-DDPP. One provider could not provide usage data, thus the present study analyses data from three NHS-DDPP providers.

### 2.2 NHS-DDPP intervention

The NHS-DDPP is a nine-month digital behaviour change intervention introduced in 2019 that focuses on improving diet, increasing physical activity and achieving weight loss, with the aim of reducing T2DM risk. The NHS-DDPP is a national intervention that commissions providers to deliver the programme, based on a standard NHS England service specification (NHS England, 2019) that specifies the overarching intervention features that should be present in the programme. Providers delivered their own versions of the digital programme, in line with the NHS England service specification (NHS England, 2019).

The service specification stipulated that the programme should include key intervention features such as goal setting, self-monitoring, educational content, and social support (NHS England, 2019; Hawkes et al., 2022a). However, the modalities in which providers delivered these intervention features differed, summarised in Table 1. The first three months of the NHS-DPP are considered the ‘core’ phase of the programme, and months 4–9 the ‘maintenance’ phase where support gradually tapers off. Further information on the NHS-DDPP is provided elsewhere (Miles et al., 2023a; Hawkes et al., 2023).

**Table 1:** Summary of main features of NHS-DDPP provider programme delivery, highlighting differences between providers.

	Provider A	Provider C	Provider D
Self-monitoring	Mode of delivery: app	Mode of delivery: app	Mode of delivery: app
	Users could track the following behaviours in the app: diet, physical activity, fluid intake, mood, appetite, bowel movements, symptoms.	Users could track the following behaviours in the app: diet, physical activity, steps, sleep, alcohol, smoking, medicine, pain, mood.	Users could track the following behaviours in the app: diet, steps, sleep.
	Users could track the following outcomes in the app: weight, blood glucose levels, blood pressure, waist circumference.	Users could track the following outcomes in the app: weight, blood glucose levels, fasting blood sugar, blood pressure, waist-hip ratio.	Users could track the following outcomes in the app: weight.

Goal setting	Mode of delivery: app	Mode of delivery: app	Mode of delivery: app
	Users could set goals for the following behaviours in the app: diet, physical activity, fluid intake, mood, appetite, bowel movements, symptoms.	Users could set goals for the following behaviours in the app: diet, physical activity, steps, sleep, alcohol, smoking, medicine, pain, mood.	Users could set goals for the following behaviours in the app: diet, physical activity, mind-set, stress, sleep, custom (free text).
	Users could set goals for the following outcomes in the app: weight, blood glucose levels, blood pressure, waist circumference.	Users could set goals for the following outcomes in the app: weight, blood glucose levels, fasting blood sugar, blood pressure, waist-hip ratio.	Users could set goals for the following outcomes in the app: weight.

Educational content	Mode of delivery: articles via app, also via online learning platform	Mode of delivery: articles/videos/links sent by health coach via message in app	Mode of delivery: articles via app, also via online learning platform
	42 lessons comprising articles which included text, images, videos, podcasts and links to external websites.	Tailored educational articles, videos and website links sent from health coach to service users via message in app.	Articles which included text, images, videos, podcasts and links to external websites.
	Content unlocked weekly throughout nine-months.	Content is sent weekly (months 1–3), bi-weekly (months 4–6), and monthly (months 7–9) over the nine-month programme.	Content unlocked weekly during the first three months of the programme. Seven optional ‘Sustain’ courses through months 4–7 of the programme with more in-depth information on education topics.
	All providers covered topics such as dietary fibre, alcohol, physical activity, managing stress, sleep, and managing social events.		

Social support	Mode of delivery: health coach support via calls and messaging in app	Mode of delivery: health coach support via calls and messaging in app	Mode of delivery: health coach support via messaging in app
	Monthly telephone call to discuss progress and review goals. Health coaches send messages to users and provide feedback on behaviours/outcomes tracked in the app.	Initial 45-minute video call to discuss programme and set goals. Regular messages from health coach throughout nine-month programme, including receiving educational content and feedback on behaviours/outcomes tracked in the app. Health coaches check-in with users weekly, then bi-weekly and then monthly.	Support from health coach via the group chat during the first three months of the programme. One-to-one messaging also available during first three months of programme.
		Mode of delivery: group support in app	Mode of delivery: group support in app
		Open group discussion forum available throughout the nine-month programme.	Users allocated to a closed group chat (10–15 people, available on app), moderated by a health coach for the first three months of the programme. Closed group is available through months 4–9 without the health coach moderation.
			Open group discussion forum available in months 4–9.

Note. Provider B was unable to supply usage data for this analysis. Providers are labelled A, C and D to provide anonymisation, but to allow cross-reference to previous papers published by the research team for this programme of work (Hawkes et al., 2022a; Hawkes et al., 2023).

Note. Provider A did not offer group support at the time this study was conducted, but now offers a group support pathway to users who wish to receive group support via a group chat.

### 2.3 Participants

Service users took part in one of the three digital programmes, depending on which provider was commissioned to deliver the digital service in their local geographical area at the time of enrolment. Service users were 18 years and over, living in England and identified as being high risk for developing T2DM. They were either referred onto the programme via their general practice if their records indicated them to have non-diabetic hyperglycaemia (defined as HbA1c in the range of 42–47 mmol/mol [6.0–6.4 %]) or via an online self-referral questionnaire (Diabetes UK, 2020b). This questionnaire included questions on age, gender, ethnicity, waist circumference and bodyweight. If the questionnaire deemed individuals to be at medium or high risk, they could self-refer onto the programme. Service users were not eligible if they were pregnant or under 18 years old.

### 2.4 Procedures

Researchers were in contact with the management staff employed by each of the providers to agree on the usage data fields they were able to share with the research team. We asked for specific data fields that related to time spent on the app, BCT usage (e.g., goal setting, self-monitoring), and other engagement measures such as access to educational content, support from a health coach and group forum usage. An opt-out consent method for service users was agreed with each provider, consisting of an information statement included in each of the providers' terms and conditions/privacy policies when service users signed up to download the provider apps. This approach ensured that participants were not likely to react to the assessment of usage data by changing their behaviours (French et al., 2021).

### 2.5 Measures

‘Effective engagement’ (Miller et al., 2019) was defined as engagement with key intervention features designed to help people improve their diet, increase their physical activity and achieve weight loss. In the NHS-DDPP, these included self-monitoring, goal setting, receiving educational content (via educational articles), and social support (via health coach and group forum). This was based on the key components specified for inclusion in the NHS-DDPP (NHS England, 2019), and identified in the researcher-developed NHS-DPP logic model (Hawkes et al., 2021).

In line with the AMUsED framework (Miller et al., 2019), analytic indicators focused on the frequency of interactions with different intervention features over the nine-month programme (Pham et al., 2019) (See Appendix 1). Given that this was an independent evaluation of a national programme already implemented in practice at the time of data collection, the research team were restricted to the data fields that the digital providers were able to share with them (e.g., providers might not routinely collect some data on intervention features requested by the research team). Thus, data fields varied across each provider (see Table 2 for a summary of engagement measures obtained from each provider and see Appendix 2 for definitions of data fields supplied by providers).

**Table 2:** Summary of engagement measures included in usage data analysis across providers.

	Provider A	Provider C	Provider D
Self-monitoring^a^			
Number of times behaviours were self-monitored	✓	✓	✓
Number of times outcomes were self-monitored	✓	✓	✓

Goal setting^a^			
Number of times a behavioural and/or outcome goal was set or amended	✓	✓	✓
Number of times behavioural goals were set	✗	✓	✓
Number of times outcome goals were set	✗	✓	✓

Educational content			
Number of times any articles were accessed	✓	✗	✓
Number of times unique articles were accessed	✓	✗	✓
Number of times educational content was sent from health coach	n/a	✓	n/a

Health coach support			
Number of calls with health coach	✓	✓	n/a
Number of messages sent to health coach that have been responded to	✓	✗	✓
Number of messages sent from service user to health coach (including text, images and videos)	✗	✓	✗
Number of support messages sent from health coach (including text, images, links and videos)	✗	✓	✗

Group support			
Number of peer messages sent in group chat by service user^b^	n/a	n/a	✓
Number of group posts in discussion forum by service user^c^	n/a	✓	✓
Number of comments on group posts in discussion forum by service user^c^	n/a	✓	✓
Number of likes on group posts in discussion forum by service user^c^	n/a	✓	✓

App usage			
Number of minutes spent on the app^d^	✓	✓	✓

Note. Provider B was unable to supply usage data for this analysis. Providers are labelled A, C and D to provide anonymisation, but to allow cross-reference to previous papers published by the research team for this programme of work (Hawkes et al., 2022a; Hawkes et al., 2023).

a If users self-monitored more than one behaviour/outcome during one individual occasion, or set a goal for more than one behaviour/outcome during one individual occasion, this was reported as individual instances of self-monitoring or goal setting.

b Closed peer group chat messaging, also moderated by a health coach.

c Open group discussion forums, akin to social media forum.

d This measure includes time spent using the intervention features in the app and is therefore considered an overall measure of engagement. ✓ = Provider has supplied this data. ✗ = Provider has not supplied this data but it is part of their digital intervention. n/a = Not offered as part of the provider's digital intervention at the time of the evaluation.

### 2.6 Data analyses

Anonymous usage data was received from providers in Excel spreadsheets. Two providers sent usage data for each user which was already aggregated into 30-day ‘engagement periods’ (in line with how NHS-DDPP commissioners measure engagement) over the nine months, and one provider sent individual-level usage data detailing time-stamped information on what anonymised individual users had engaged with across the nine-month intervention. All datasets were cleaned in Excel and individual-level usage data was aggregated into nine 30-day ‘engagement periods’. Usage data were divided into these nine engagement periods to understand how usage changed across the duration of the programme for each of the engagement measures listed in Table 2. Given that the NHS-DDPP was a national programme, usage was first described for the whole NHS-DDPP, and then compared across providers who delivered their own versions of the programme.

Descriptive statistics (means, medians and ranges) were calculated in SPSS to describe the dose of app usage across the nine-month programme, as well as across engagement periods and by providers. Given that data were not normally distributed, non-parametric tests were used to assess differences across providers.

### 2.7 Ethics approval

This study was reviewed and approved by the North West Greater Manchester East NHS Research Ethics Committee (Reference: 17/NW/0426, 1st August 2017).

## 3 Results

Usage data for the nine-month programme was obtained for a total of 1826 service users across the three digital providers. Provider A supplied data for *n* = 940 service users starting the NHS-DDPP between 20/05/2021 and 19/06/2021. Providers C and D supplied data for *n* = 283 (provider C) and *n* = 603 (provider D) service users starting the NHS-DDPP between 01/12/2020 and 28/02/2021. The date range for the cohort of service users differed across providers as this was dependent on when an opt-out consent procedure was in place for each provider.

### 3.1 Duration of engagement on the NHS-DDPP

App usage decreased over time from a median of 32 min (IQR 191) in engagement period 1 to 0 min (IQR 14) in engagement period 9. A total of 1230 users (67 %) had spent at least 1 min on the app during engagement period 1, compared to 1008 users (55 %) in engagement period 5, and 667 users (37 %) in engagement period 9. See Table A1 in Appendix 3 for percentages of users who spent at least 5 and 10 min in the app during those engagement periods.

**Fig. 1:**
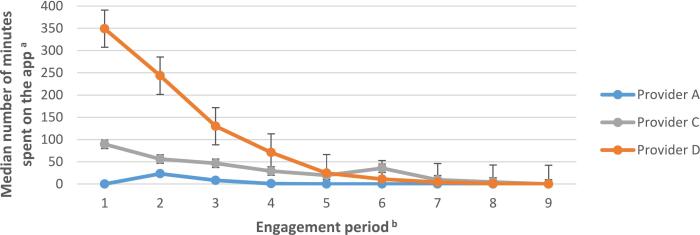
Median number of minutes spent on the app across the nine-month programme. *Note*. n(provider A) = 940; n(provider C) = 283; n(provider D) = 603. *Note*. The educational content for providers A and D could be accessed both via the app and via a website. Only when educational content was accessed via the app this was registered as time spent on the app. *Note*. Post-hoc Mann-Whitney U tests showed that service users on provider D’s programme had spent significantly more time on the app during engagement period 1 compared to service users on provider C’s programme (*U*[*N*provider C = 283, *N*provider D = 603] = 43479.5, *p* < 0.001), who had spent significantly more time on the app during engagement period 1 compared to those on provider A’s programme (*U*[*N*provider A = 940, *N*provider C = 283] = 25634.5, *p* < 0.001). ^a^ Across the three providers, the time spent on the app was only registered when service users spent ≥ 30 seconds on the app. If users completed the required app functions in under 30 seconds, this usage was not registered in the current dataset. ^b^ Engagement period denotes 30 day periods: engagement period 1 = days 1 to 30; engagement period 2 = days 31 to 60, etc.

There was substantial variation in app usage across the different providers (Fig. 1). Median usage in the first engagement period was 349 min (IQR 701) for users enrolled with provider D, compared to 90 min (IQR 114) for provider C and 0 min (IQR 13) for provider A. However, usage sharply declined for provider D during the first 4 months of the programme. A Kruskal Wallis test showed significant differences between providers in the amount of time users spent on the app during engagement period 1 (*H*(2) = 1103.9, *p* < 0.001).

### 3.2 Overall engagement with NHS-DDPP intervention features across the nine-month intervention

Overall, service users primarily used the apps for self-monitoring their behaviours, with these features accessed a median of 117 times across the nine-month programme (see Table 1 and Appendix 2 for the behaviours monitored as part of each provider's service provision), and accessing educational articles (accessed a median of 52 times) (Table 3). The open group discussion forums were engaged with the least frequently across the NHS-DDPP (accessed a median of 0 times across the programme), see Table 3. Notably, 610 of the 886 users (68.8 %) with access to an open group discussion forum did not engage with these features at all. Conversely, those who had access to peer support via a smaller closed group chat offered by one provider engaged with this feature a median of 6 times. The mean number of times apps were accessed was generally higher than the median values, indicating that there were some very active users whilst a significant proportion barely engaged at all.

**Table 3:** Overall frequency of use of features of the NHS-DDPP.

	Frequency of use across 9-month programme		
	*n*	Mean (SD)	Median (IQR)
Self-monitoring^a^			
Number of times behaviours were self-monitored	1826	384.4 (686.8)	117.0 (451.0)
Number of times outcomes were self-monitored	1826	20.8 (49.1)	6.0 (20.0)

Goal setting^a^			
Number of times a behavioural and/or outcome goal was set or amended	1826	114.5 (286.7)	6.0 (55.0)
Number of times a behavioural goal was set	886	4.1 (5.4)	2.0 (5.0)
Number of times an outcome goal was set	886	0.9 (1.4)	0.0 (1.0)

Educational content			
Number of times articles were accessed	1543	128.1 (217.3)	52.0 (165.0)
Number of times unique articles were accessed	1543	64.3 (81.4)	32.0 (75.0)
Number of times educational content was sent from health coach	283	32.5 (17.0)	36.0 (26.0)

Health coach support			
Number of calls with health coach	1223	2.6 (2.7)	1.0 (5.0)
Number of messages sent to health coach that have been responded to	1543	25.5 (114.5)	7.0 (23.0)
Number of messages sent from service user to health coach	283	20.8 (8.0)	23.0 (10.0)
Number of support messages sent from health coach	283	27.7 (56.9)	15.0 (23.0)

Group support			
Number of peer messages sent in group chat by service user^b^	603	29.8 (79.3)	6.0 (31.0)
Number of group posts in discussion forum by service user^c^	886	0.2 (1.7)	0.0 (0.0)
Number of comments on group posts in discussion forum by service user^b^	886	2.6 (40.9)	0.0 (0.0)
Number of likes on group posts in discussion forum by service user^c^	886	5.0 (32.4)	0.0 (1.0)

Note. n(provider A) = 940; n(provider C) = 283; n(provider D) = 603.

Note. SD denotes standard deviation; IQR denotes inter-quartile range.

a If users self-monitored more than one individual behaviour/outcome during one occasion, or set a goal for more than one individual behaviour/outcome during one occasion, this was reported as individual instances of self-monitoring or goal setting.

b Closed peer group chat messaging, also moderated by a health coach.

c Open discussion group forums, akin to social media forum.

### 3.3 Engagement with intervention features over time

Engagement with intervention features of the NHS-DDPP decreased across the nine-month programme (see Table 4), though a minority of service users did engage with features throughout the duration of the programme (see ranges in Table 4). By month 4, over half of users were no longer engaging with any of the features of the digital programme, with the exception of self-monitoring behaviours (median = 1) and sending messages to their health coach (median = 1).

**Table 4:** Engagement with features of NHS-DDPP across the nine-month intervention (median, IQR, range).

	*n*	Engagement period^a^								
		1	2	3	4	5	6	7	8	9
		Median (IQR) [Range]								
Self-monitoring^b^										
Number of times behaviours were self-monitored	1826	35 (87) [0–3214]	28 (87) [0–1483]	10 (72) [0–680]	1 (55) [0–659]	0 (36) [0–68]	0 (29) [0–667]	0 (16) [0–661]	0 (8) [0–648]	0 (1) [0–662]
Number of times outcomes were self-monitored	1826	2 (6) [0–85]	1 (5) [0–147]	1 (4) [0–116]	0 (3) [0–98]	0 (0) [0–138]	0 (0) [0−102]	0 (0) [0–64]	0 (0) [0–241]	0 (0) [0−113]

Goal setting^b^										
Number of times a behavioural and/or outcome goal was set or amended	1826	4 (18) [0–319]	0 (12) [0−300]	0 (7) [0–300]	0 (3) [0–300]	0 (1) [0–300]	0 (0) [0–300]	0 (0) [0–300]	0 (0) [0–256]	0 (0) [0–261]
Number of times a behavioural goal was set	886	2 (4) [0–27]	0 (1) [0−12]	0 (0) [0−13]	0 (0) [0–8]	0 (0) [0–8]	0 (0) [0–7]	0 (0) [0–5]	0 (0) [0−10]	0 (0) [0–5]
Number of times an outcome goal was set	886	0 (1) [0–10]	0 (0) [0–4]	0 (0) [0–3]	0 (0) [0–4]	0 (0) [0–4]	0 (0) [0–2]	0 (0) [0–9]	0 (0) [0–4]	0 (0) [0–2]

Educational content										
Number of times articles were accessed	1543	12 (48) [0–846]	5 (36) [0–636]	2 (18) [0–400]	0 (8) [0–335]	0 (1) [0–385]	0 (3) [0–316]	0 (4) [0−201]	0 (4) [0–197]	0 (4) [0–249]
Number of times unique articles were accessed	1543	6 (23) [0–119]	4 (23) [0–125]	2 (14) [0−123]	0 (4) [0–150]	0 (0) [0–90]	0 (2) [0–143]	0 (4) [0–89]	0 (4) [0–197]	0 (3) [0–214]
Number of times educational content was sent by health coach	283	8 (5) [0−20]	6 (4) [0–13]	6 (5) [0–17]	4 (4) [0–10]	4 (5) [0–12]	2 (4) [0–14]	1 (2) [0–17]	1 (2) [0–10]	0 (2) [0–18]

Health coach support										
Number of calls with health coach	1223	1 (1) [0–3]	0 (0) [0–4]	0 (0) [0–2]	0 (1) [0–2]	0 (1) [0–2]	0 (1) [0–3]	0 (0) [0–2]	0 (0) [0–2]	0 (0) [0–2]
Number of messages health coach has responded to	1543	1 (7) [0–185]	1 (9) [0−323]	0 (3) [0–339]	0 (1) [859]	0 (0) [0–802]	0 (0) [0–280]	0 (0) [0−302]	0 (0) [0–518]	0 (0) [0–513]
Number of messages sent from health coach	283	5 (1) [0−11]	4 (1) [0–9]	4 (2) [0–14]	2 (3) [0–10]	2 (1) [0–6]	2 (3) [0–7]	1 (2) [0–5]	1 (1) [0–4]	0 (1) [0–4]
Number of messages sent from service user	283	4 (6) [0–191]	2 (4) [0–135]	2 (5) [0–102]	1 (3) [0–146]	1 (2) [0−131]	0 (2) [0–46]	0 (1) [0–35]	0 (1) [0−30]	0 (1) [0–30]

Group support										
Number of peer messages sent in group chat by service user	603	2 (11) [0–152]	1 (9) [0–246]	0 (5) [0–285]	0 (2) [0–470]	0 (0) [0–259]	0 (0) [0–68]	0 (0) [0–40]	0 (0) [0−22]	0 (0) [0−31]
Number of group posts in discussion forum by service user	886	0 (0) [0–12]	0 (0) [0–2]	0 (0) [0–1]	0 (0) [0–6]	0 (0) [0–10]	0 (0) [0–13]	0 (0) [0–4]	0 (0) [0–6]	0 (0) [0–9]
Number of comments on group posts in discussion forum by service user by service user	886	0 (0) [0–13]	0 (0) [0–30]	0 (0) [0–75]	0 (0) [0−220]	0 (0) [0–189]	0 (0) [0–58]	0 (0) [0–175]	0 (0) [0–278]	0 (0) [0–274]
Number of likes on group posts in discussion forum by service user	886	0 (0) [0–48]	0 (0) [0–49]	0 (0) [0–177]	0 (0) [0–64]	0 (0) [0–79]	0 (0) [0–107]	0 (0) [0–98]	0 (0) [0–173]	0 (0) [0–196]

Note. The numbers in curved brackets denote the inter-quartile range (IQR). The numbers in squared brackets denote the range.

Note. n(provider A) = 940; n(provider C) = 283; n(provider D) = 603.

a Engagement period denotes 30 day periods: engagement period 1 = days 1 to 30; engagement period 2 = days 31 to 60, etc.

b If users self-monitored more than one individual behaviour/outcome during one occasion, or set a goal for more than one individual behaviour/outcome during one occasion, this was reported as individual instances of self-monitoring or goal setting.

### 3.4 Differences in engagement with intervention features across provider programmes

As each provider delivered their own versions of the NHS-DDPP, the differences in engagement in intervention features across provider programmes are described below. See Table A2 in Appendix 4 detailing intervention feature use across nine-month programme, broken down by provider.

#### 3.4.1 Use of self-monitoring functions on the NHS-DDPP

**Fig. 2:**
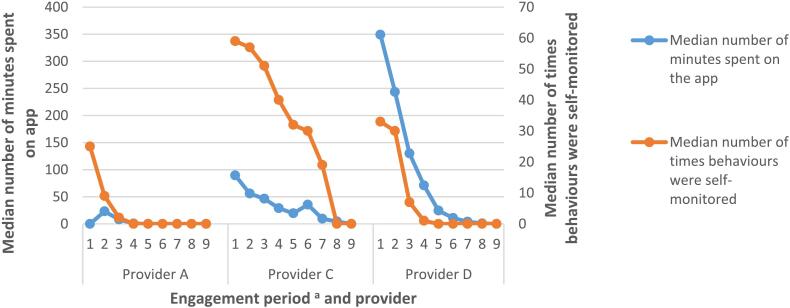
Engagement with self-monitoring of behaviours across the nine-month programme, compared with the time spent on the app. *Note*. n(provider A) = 940; n(provider C) = 283; n(provider D) = 603. *Note*. If users self-monitored more than one behaviour/outcome during one individual occasion, this was reported as individual instances of self-monitoring. ^a^ Engagement period denotes 30 day periods: engagement period 1 = days 1 to 30; engagement period 2 = days 31 to 60, etc.

Usage of self-monitoring of behaviours decreased over time across providers (see Table A2 in Appendix 4). Outcomes of behaviour (e.g., weight) were monitored significantly less on the apps, though users on provider D's programme used this function until engagement period 4 (Appendix 4). Service users on provider C's programme more frequently self-monitored their behaviours on the app throughout the duration of the programme compared to providers A and D (see Fig. 2).

#### 3.4.2 Use of goal setting functions on the NHS-DDPP

**Fig. 3:**
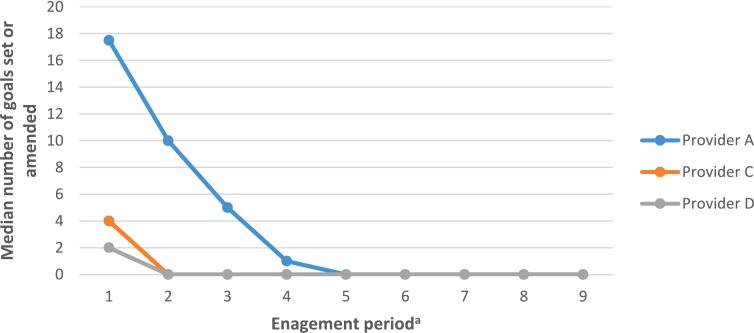
Median number of goals set or amended across the nine-month programme. *Note*. n(provider A) = 940; n(provider C) = 283; n(provider D) = 603. *Note*. If users set a goal for more than one behaviour/outcome during one individual occasion, this was reported as individual instances of goal setting. ^a^ Engagement period denotes 30 day periods: engagement period 1 = days 1 to 30; engagement period 2 = days 31 to 60, etc.

Those on provider A's programme set or amended goals more frequently throughout the nine-months (see Fig. 3 and Table A2 in Appendix 4). In comparison, users on provider C and D's programmes primarily set a goal at the start of the programme, with no further engagement with goal setting.

#### 3.4.3 Engagement with educational content on the NHS-DDPP

**Fig. 4:**
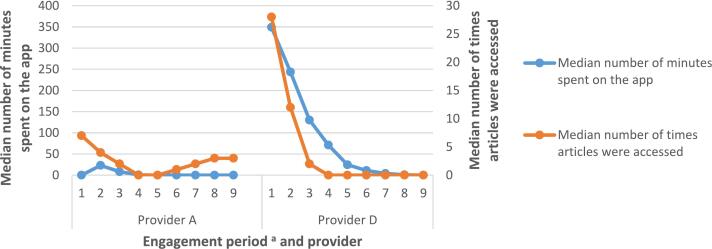
Access of educational articles across nine-month programme for providers A and D. *Note*. n(provider A) = 940; n(provider D) = 603. *Note*. The educational content for providers A and D could be accessed both via the app and via a website. Only when educational content was accessed via the app this was registered as time spent on the app. ^a^ Engagement period denotes 30 day periods: engagement period 1 = days 1 to 30; engagement period 2 = days 31 to 60, etc.

Those on provider D's programme accessed articles more frequently during the first three months of the programme (median = 28, 12 and 2 times during engagement periods 1–3 respectively), before starting the ‘maintenance’ phase of the programme (engagement periods 4–9, where users could enroll onto optional courses or revisit topic areas; median = 0 times). This correlated with time spent on the app (see Fig. 4). Users on provider A's programme accessed materials throughout the nine-month programme (median = 7 articles accessed in engagement period 1 compared to 3 articles accessed in engagement period 9). See Table A2 in Appendix 4.

**Fig. 5:**
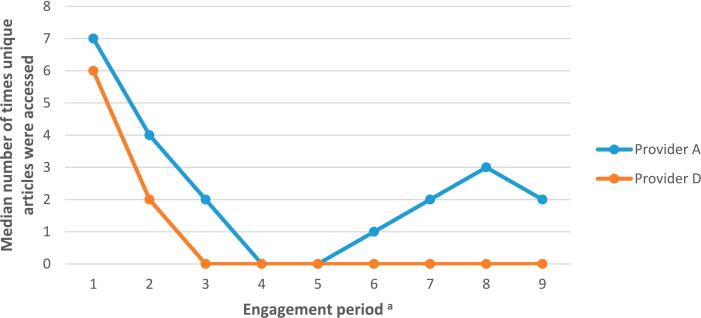
Access of unique educational articles across nine-month programme for providers A and D. *Note*. n(provider A) = 940; n(provider D) = 603. *Note*. The educational content for providers A and D could be accessed both via the app and via a website. Only when educational content was accessed via the app this was registered as time spent on the app. ^a^ Engagement period denotes 30 day periods: engagement period 1 = days 1 to 30; engagement period 2 = days 31 to 60, etc.

Those on provider A's programme accessed a higher number of unique educational articles throughout the nine-month programme compared to users on provider D's programme (Fig. 5). For example, users on provider A's programme accessed a median of 2 unique educational articles during engagement period 9 compared to a median of zero for users on provider D's programme (see Fig. 5 and Appendix 4).

#### 3.4.4 Use of support provided on the NHS-DDPP

The number of peer messages sent via the group chats (offered by provider D) during the first three months were higher (median = 2 and 1 peer messages sent for engagement periods 1 and 2 respectively), compared to the number of times the group discussion forums were used on the programmes of providers C and D (medians = 0 across all engagement periods for both providers; see Table A2 in Appendix 4).

Table 5 compares the user engagement findings from this study to the differences in provider programmes. When health coach support was linked to specific intervention features, this was associated with higher engagement with that intervention feature.

**Table 5:** User engagement differences between providers and associated differences in provider programme delivery.

	Provider A	Provider C	Provider D
Self-monitoring			
User engagement differences		Users more frequently self-monitored their behaviours on the app and had continued engagement with self-monitoring features over the nine-month duration.	
Provider programme features		Health coaches provided tailored feedback on tracked behaviours and outcomes via one-to-one messaging.	

Goal setting			
User engagement differences	Users set or amended goals more frequently throughout the nine-months.		
Provider programme features	Health coaches reviewed goals and progress with users via telephone calls and one-to-one messaging.		

Educational content			
User engagement differences	Users accessed a higher number of unique educational articles throughout the 9-month programme.		Users accessed more educational articles during the first 3-months of the programme.
Provider programme features	Educational content was unlocked weekly over 9-months.		Educational content was unlocked weekly during the first 3-months.

Group support			
User engagement differences			Users sent more peer messages via the group chat during the first 3-months of the programme. Open group support forums rarely used.
Provider programme features			Closed group chats were moderated by a health coach during the first 3-months.

Note. A fuller description of provider programme features is reported in Table 1, and more detailed user engagement data broken down by provider is reported in Table A2 of Appendix 4.

## 4 Discussion

### 4.1 Principal findings

A decrease in app usage was observed over the nine-month programme, although the rate at which the decrease occurred varied substantially between providers. Service users primarily used the apps to self-monitor their behaviours, and to access educational content. Group support functions were not used regularly, though there were some differences in use of group chats versus group discussion forums offered by provider programmes. Engagement with intervention features was higher when health coach support was linked to those specific elements of the programme.

### 4.2 Strengths and limitations

This novel study presents routinely collected data from a nationally implemented programme, rather than trial data, thus providing a snapshot of how users engaged with a digital behaviour change programme in a real-world setting. The present analysis compared usage data longitudinally across the nine-month intervention, providing much greater context on service users' needs compared to just the total amount of engagement across groups (Pham et al., 2019). Analysis of the different features used in the NHS-DDPP allowed further understanding of the breadth of NHS-DDPP app engagement.

Analysis of usage data from three different providers was a further strength; not only is analysis of digital interventions in the real world rare, but linking differences in engagement between providers to variation in programme contents allows identification of intervention features that may promote engagement. Evaluations of interventions produced via this model of commissioning are also rare, thus the present work may provide a useful exemplar for future evaluations. We used a structured framework (Miller et al., 2019) to guide a systematic and fine-grained analysis of usage data across the NHS-DDPP providers. This identified key intervention features (e.g., self-monitoring, goal setting, receiving educational content, social support) as the meaningful variables to include in the usage data analysis, as in line with the previously developed logic model for the programme (Hawkes et al., 2021).

Despite substantial efforts, we were unable to obtain usage data from one of the NHS-DDPP providers, and the data obtained from other providers differed according to provider reporting capabilities and specific intervention features. This limitation demonstrates the logistical issues of obtaining usage data from a live national programme with independent digital providers. Working with real-world data necessarily requires combining the functionality of different datasets from different providers, however we used theory to compare usage of intervention features even when there were differences in data capture.

This analysis sheds light on what intervention features service users engaged with on the NHS-DDPP, but it cannot tell us why there was disengagement with the apps, and the lack of demographic data meant that we could not assess disparities in digital access that might have influenced engagement. Further, we were unable to obtain outcome data for the present analysis (e.g., bodyweight, HbA1c), so we were therefore unable to establish whether usage of NHS-DDPP intervention features is associated with weight loss or improved blood glucose levels.

### 4.3 Comparison with prior work

Engagement with the NHS-DDPP in the present study follows a similar usage pattern seen in trials of digital diabetes prevention programmes, including patterns of attrition (Harjumaa et al., 2020; Lavikainen et al., 2022) and the extent and duration of which users engage with different intervention features (Block et al., 2015). Drawing direct comparisons with previous interventions studied in trials are somewhat problematic due to differences in intervention dosage. However, the present findings suggest that patterns of usage in this target population in a nationally implemented intervention are similar to those observed in a trial sample. Previous research assessing engagement with the face-to-face NHS-DPP found that 34 % of service users completed 60 % of the programme, and 22 % of service users went on to complete the full course (Howarth et al., 2020). Thus, engagement with the digital programme compares favourably. This corresponds with trial data which found participation to be higher in a digital diabetes prevention programme compared to the face-to-face alternative (Moin et al., 2018).

The present research suggested that engagement with some intervention features were higher when health coach support was linked to those specific features. For example, the provider programme that offered monthly telephone calls with a health coach where users could review their progress was also the programme where users set and amended the highest number of goals. This highlights the health coach's role in keeping users engaged and accountable (Yardley et al., 2016). Interviews with users on the NHS-DDPP found that health coaches provided them with person-centred support, and coaching was instrumental in helping them to understand and use BCTs such as goal setting and receiving feedback on behaviours they had monitored (Miles et al., 2023b), which could explain the current findings. Previous research also found that interaction with health coaches increased engagement with self-monitoring and was associated with subsequent weight loss in a digital diabetes prevention programme (Painter et al., 2020). Both results are in line with systematic reviews which have found that human-delivered support can positively influence engagement with digital behaviour change interventions (Perski et al., 2017), promote weight loss (Chew et al., 2022; Antoun et al., 2022), and increase adherence with mHealth app interventions (Jakob et al., 2022).

However, users were not engaging with the open group discussion forums on the NHS-DDPP. On the one hand, evidence has found group support to be a predictor of weight loss in digital diabetes prevention programmes (Sepah et al., 2017; Michaelides et al., 2016; Michaelides et al., 2018). Additionally, access to peer support was associated with a significantly greater weight reduction at 12 months in a recent evaluation of the pilot NHS-DDPP (Ross et al., 2022). On the other hand, people on the NHS-DDPP report to opt for digital programmes because they do not like the group aspect (Ross et al., 2023), and these larger online networks (akin to social media) may lack the ‘critical mass’ to deliver sufficient personalised support (Elaheebocus et al., 2018). This may explain why users on the provider programme who offered closed peer group support moderated by a health coach showed higher engagement with this intervention feature in the current analysis.

### 4.4 Implications

We found that users typically reduced engagement with the NHS-DDPP over the nine-month programme, though there were a minority of users who utilised features of the apps frequently throughout the intervention. The current study cannot tell us why there was a reduction in usage, but based on previous understanding of user engagement with digital health interventions there are two possible reasons: (a) users may have disengaged with the programme due to a lack of satisfaction with the intervention features, or (b) users may have got what they wanted from the programme, e.g., they had developed habits to continue maintaining their lifestyle changes and no longer felt they needed the app (Yardley et al., 2016). However, there was higher engagement with some intervention features (e.g., goal setting) when support from a health coach was linked to those features, suggesting that users may see some merit in continuing to use the intervention features if support is provided.

Thus far, an assessment of the extent to which the NHS-DDPP has been delivered as intended has demonstrated that fidelity to the programme specification stipulated by NHS England (NHS England, 2019) is better than for the face-to-face programme (Hawkes et al., 2022b). This might be expected, as staff do not have to be trained to deliver *all* the intervention content. However, data from this analysis and results from the wider programme of research (Hawkes et al., 2023; Miles et al., 2023b) suggest that some human element may still be necessary in digital behaviour change programmes to improve engagement with self-regulatory processes such as goal setting. Intervention developers and commissioners of behaviour change programmes should consider this when designing and commissioning digital programmes. Although employing and training staff to deliver some aspects of digital behaviour change interventions would incur additional cost, this may yield higher engagement with key intervention features, improve participant understanding and experience (Miles et al., 2023b), and thereby increase the effectiveness of the intervention. This warrants further research examining the impacts of engagement on effectiveness (e.g., weight loss) and an economic assessment of the associated costs.

The evidence underpinning the development of the NHS-DPP suggests that diabetes prevention programmes are most effective when programme length was over six months compared to a programme duration of less than three months (Ashra et al., 2015). However, this evidence was based on reviews of face-to-face interventions (Yardley et al., 2016). This study has shown that the largest decrease in engagement with the NHS-DDPP was after three months, which also coincided with when providers reduced their contact with users. Future research should assess outcomes of those on the NHS-DDPP (e.g., HbA1c and bodyweight), and compare the outcomes of those who disengaged with those who continued to engage with the programme. This could establish whether a drop-off in engagement is due to users having achieved health outcomes and would thus indicate whether a shorter programme duration might be suffice, or whether providers should be encouraged to increase their interactions with users at a later stage during the programme to maintain engagement for a longer period.

The lack of engagement with group support in the current study might indicate that users are missing out on a key intervention component. The provider that offered closed groups chats moderated by health coaches elicited higher engagement in the NHS-DDPP compared to usage of open group discussion forums. We do not know from this study whether it is the closed nature of the groups that elicited higher engagement, the moderation from a health coach, or both. More research is needed to assess whether certain types of group support (e.g., open discussion forums vs. closed group chats) optimise engagement and effectiveness of digital behaviour change programmes, which would further establish whether this component is key for intervention effectiveness.

To further understand real-world behavioural intervention usage, future studies that compare ‘live’ interventions should engage in data linkage as early as possible such that collected data can be meaningfully aggregated across service providers. Finally, given that obtaining outcome data (e.g., bodyweight) was not possible for the current analysis, future evaluation research for this national programme should examine the dose response in relation to participants' adherence with intervention features of the NHS-DDPP and the impact this has on outcomes (e.g., weight loss, HbA1c).

### 4.5 Conclusions

This study analysed usage data from a cohort of service users enrolled on the nationally-implemented English digital diabetes prevention programme, providing insights into how users engage with a digital programme in a real-world setting across the nine-month intervention duration. The rate of the decrease in app usage varied substantially, with engagement varying both between individuals and across programme providers. Health coach support that was linked to specific features (e.g., goal setting and self-monitoring) was associated with higher engagement with that intervention feature. Future research is needed to compare engagement rates and outcomes, both within and across providers.

## Abbreviations

BMI: Body Mass Index
HbA1c: Hemoglobin A1c
NHS-DPP: National Health Service Diabetes Prevention Programme
NHS-DDPP: National Health Service Digital Diabetes Prevention Programme
T2DM: Type 2 diabetes mellitus
UK: United Kingdom
BCT: Behaviour Change Technique
AMUsED: Analyzing and Measuring Usage and Engagement Data

## Ethics approval and consent to participate

The wider programme of research of which this study is a part of was reviewed and approved by the North West Greater Manchester East NHS Research Ethics Committee (Reference: 17/NW/0426, 1st August 2017). Full verbal consent was obtained from all participants included in this study.

## Consent for publication

Not applicable.

## Availability of data and materials

The anonymous engagement data from the current study are not publicly available due to confidentiality agreements with the provider organisations. Some datasets are available from the corresponding author on reasonable request, although authors will require the explicit permission of the relevant provider organisations.

## Funding

This work is independent research funded by the National Institute for Health and Care Research (The Health and Social Care Delivery Research (HSDR) Programme, 16/48/07 – Evaluating the NHS Diabetes Prevention Programme (NHS DPP): the DIPLOMA research programme (Diabetes Prevention – Long Term Multimethod Assessment)). The views and opinions expressed in this manuscript are those of the authors and do not necessarily reflect those of the National Institute for Health and Care Research or the Department of Health and Social Care.

## CRediT authorship contribution statement

DPF designed the research and secured funding for it as part of the wider DIPLOMA project. DPF supervised the research conduct, helped interpret the data and helped prepare the manuscript. REH elicited the data from service providers, cleaned the data, analysed and interpreted the data, and prepared the manuscript. LMM elicited the data from service providers, helped interpret the data, and helped to draft the manuscript. JAR and BA helped interpret the data and helped draft the manuscript. RM secured funding for the research as part of the wider DIPLOMA project, helped interpret the data, and contributed to the drafting of the manuscript. All authors read and approved the final manuscript.

## Declaration of competing interest

The authors declare that they have no known competing financial interests or personal relationships that could have appeared to influence the work reported in this paper.

## Appendices 1-4 Supplementary data

Appendix 1 (.doc): Applying AMUsED framework to analysis. Appendix 2 (.doc): Usage data field definitions. Appendix 3 (.doc): Duration of engagement over the 9-month intervention. Appendix 4 (.doc): Engagement with intervention features across the 9-month intervention, broken down by provider. Image 1
