# Supplementary material for: Engagement with a nationally-implemented digital behaviour change intervention: Usage patterns over the 9-month duration of the National Health Service Digital Diabetes Prevention Programme
Source: Internet Interv. 2023 Jul 12;33:100647. doi: 10.1016/j.invent.2023.100647 (PMC10368926; doi:10.1016/j.invent.2023.100647)
Supplement: Supplementary file 1 — Appendix 1 (.doc): Applying AMUsED framework to analysis. Appendix 2 (.doc): Usage data field definitions. Appendix 3 (.doc): Duration of engagement over the 9-month intervention. Appendix 4 (.doc): Engagement with intervention features across the 9-month intervention, broken down by provider. [file mmc1.docx]

**Appendix 1: Applying AMUsED framework to analysis**

**Stage 1 checklist for the Analysing and Measuring Usage and Engagement Data (AMUsED) framework**

| **Familiarisation with the data – identifying variables** | |
| --- | --- |
| **Generic questions by data type** | |
| **1. Intervention characteristics. Data for intervention architecture and content.** | |
| **1.1. Workflow. Intervention structure and expected participant interaction and navigation through the intervention.** | |
| How many logins/sessions are available? | Apps are not structured as ‘sessions’, but users are encouraged to monitor/track their behaviours (e.g. PA, diet) and outcomes (e.g. weight), read weekly articles and users interact with their health coach (Providers A and C) or group support forum (Provider D). |
| When are they available? | Educational content gets ‘unlocked’ daily over the first three months for Provider D, and content gets unlocked weekly throughout the nine-month programme for Provider A. Material for Provider A unlocks irrespective of whether previous content has been accessed. Educational content for Provider C is only available when it is sent to the service user from the health coach during their ‘coaching interventions’.  All other content on provider apps are available at every access. |
| Are new sessions released depending on time elapsed or task-completion? | Yes for Providers A and D – educational content gets ‘unlocked’ throughout the course. |
| Are there limitations on the availability of the intervention? | No. |
| Is the purpose of a session to collect self-report measures and/or use the intervention? | No self-report measures collected (apart from tracking data). |
| When is the intervention considered to be finished? | At the end of the 9-month programme.  Continued access to the app beyond the 9 months is provided by some of the providers, but not all. |
| What prompts are used to encourage usage (e.g. emails, texts, notifications) and when are they sent? | Provider A – Users would receive an email or phone call to prompt re-engagement if they missed a contact with their health coach.  Provider C – Users receive an app reminder if they have not logged/monitored their goals for that day (e.g. logged the amount of activity they have done, or whatever their personal goals are): ‘remember to log your goals today’. Users can also receive a message that says: ‘thank you for logging your goals today’.  Text messages are sent (‘disengagement messages’) if users have not engaged with the app for a specified period of time, e.g. 7 days. |
| Does the intervention contain ‘tunnelled’ (compulsory) sequences of pages which users have to view to move forward? | Provider A – Learn content is unlocked weekly throughout the nine-month programme, irrespective of whether previous content had been accessed.  Provider D – Articles get unlocked daily for the first 3 months of the programme. |
| Are users able to select linked components they wish to view, and avoid others? | No, although users choose which goals they wish to set. Goals are personalised but components of the app remain the same for all users. |
| **1.2. Content. Content available within the pages of the intervention.** | |
| What are the components available? | Components across all provider apps:  - Users can log their personalised goals.  - Users can send messages to their health coach.  - Users can interact with a group support forum (except provider A).  - Users can log diet/PA/sleep (and other behaviours for provider C).  - Users can log their weight (and other outcomes for provider C).  - Users can view educational articles and interactive videos (e.g. exercise videos) (these are sent to those users on provider C’s programme, or available via the educational modules for providers A and D).  - Users can see automated graphs for PA and weight to show progress on steps/weight loss. |
| What is the aim of each component and are they based on underlying theoretical constructs? | Unsure of which theoretical constructs correspond to which app components; providers’ interventions are based on many theories such as self-determination theory, social cognitive theory, COM-B theory.  Researcher-developed logic model (based on the evidence base) highlights importance of self-regulatory processes (e.g. goal setting, self-monitoring) for behaviour change maintenance. |
| In what order is it anticipated the components will be used? | No specific order, though users with Providers A and C will have initial phone calls with their health coach before commencing the intervention. Provider D sends out materials via post the week prior to starting the programme (e.g. smart weighing scales, nutritional handbook). |
| What interactive features are available (e.g. forums, videos, printable information)? How long should they take to complete? | - Goal setting component – users can set personalised goals.  - Users can monitor their PA, diet, sleep and weight.  - Providers A and C – users are able to upload pictures of their foods and can receive feedback from health coach.  - One-to-one messaging with health coach.  - Providers A and D – calls with health coach.  - Providers C and D – group forum interaction.  - Users can see their activity and weight log, showing progress over the months that these have been monitored.  - Provider A – quizzes included in the educational content.  - Provider D – exercise videos contained within the app.  There is no time-frame to complete interactive features. |
| Are all components/features available to all users throughout the intervention or are some tailored for specific times or users? | - All components available, though users select their own goals that they wish to monitor throughout the programme. Goals can be amended/added at any time.  - Intervention/message content from health coach is tailored to the service user. |
| Which pages are for collecting self-report measures or for administrative purposes (e.g. questionnaires, login, password change)? | No self-report but users can change their settings (e.g. password, picture) in their settings. |
| Are there specific pages to mark the start and end of sessions? | No. |
| Which pages contain BCTs (e.g. information, planning, feedback) and what are they? | - Educational material contains a number of BCTs (as reported in the delivery analysis).  - Each of the provider apps have a home page where users can see their personalised goals, view their weekly activity data, track their weight progress over time and monitor their PA, diet, sleep and weight [**goal setting, feedback, self-monitoring**].  - Users have access to exercise videos, either present in their educational content (provider A), embedded in the ‘resources’ in the app (provider D) or sent from the health coach to the service user (provider C) [**instruction, demonstration, practice**].  - Users can engage with health coaches via one-to-one messages (providers A, C and D), via phone call (providers A and C) or can interact with group forums (providers C and D) [**social support**]. Note that BCTs from the health coach would be tailored to the users. |
| In which sessions are they available? | N/A. |
| Can specific BCTs be identified on particular pages or groups of pages? How many groups are there? | See components listed previously. |
| Do any of the pages have response options to collect information in addition to baseline/follow-up measures? What data is collected? | - Number of times users set a goal.  - Number of times users self-monitor a behaviour/outcome.  - Number of unique articles accessed (providers A and D).  - Number of articles sent to users (provider C).  - Number of posts made, comments made and likes on posts in group forum (providers C, D).  - Number of calls with health coach (provider A and C)  - Number of minutes spent on the app. |
| **2. Accrued data. Data collected during an intervention.** | |
| **2.1. Self-report. Users’ self-reported responses collected across various stages of the trial.** | |
| When are self-report questionnaires collected (e.g. weekly logins, monthly symptom information, follow-up at 6 months)? | N/A. |
| What demographic information is available (e.g. age, gender, education)? | Not available from providers. |
| Which measures are specifically related to the target behaviour and how often are they collected? | - Number of times users set a goal.  - Number of times users self-monitor a behaviour/outcome.  - Number of unique articles accessed (providers A and D).  - Number of articles sent to users (provider C).  - Number of posts made, comments made and likes on posts in group forum (providers C, D).  - Number of calls with health coach (providers A and C).  - Number of exercise videos initially accessed (provider B).  - Number of minutes spent on the app.  The above are collected any time an instance occurs, and data can be broken down into engagement periods (30-day periods) across the 9-months. |
| Which measures of beliefs influential on the target behaviour are collected and when? | N/A. |
| Are measures of health collected (e.g. conditions which may impact on target behaviour or are co-morbid) and psychosocial factors (e.g. anxiety, illness perception, motivation)? | No. |
| Are additional measures collected at follow-up (e.g. satisfaction, adherence)? | Not in the usage data. |
| **2.2. Log-data. Information automatically collected through engagement with an intervention.** | |
| What data is the software platform able to record? | Cumulative time a user spends on the app in an engagement period; Programme start date. |
| Are number, date and time of logins available by individual user? | No, only the programme start date for each user and the date that each logged event occurred. No date or time of log-ins available. |
| Are individuals’ total durations of usage accessible? | Not for individual usage sessions, but cumulative time a user spends on the app in an engagement period is documented. |
| Are the number and time of usage prompts recorded? | Data is broken down by engagement period. |
| Are there details for which pages were viewed, the sequential order and time spent viewing? | No. |
| **2.3. External data. Data collected independently but alongside intervention usage.** | |
| How and where is the data collected (e.g. GP or support staff notes, lab reports, other digital data such as activity or location trackers)? | Activity and sleep data from an external devices (e.g. Apple Watch, Fit Bit) can be linked and synced to provider apps. |
| What data is collected? | Number of steps, hours of sleep, etc. |
| Which of these measures relate to or may impact on the target behaviour? | Would impact on the number of instances an activity/sleep is measured, as captured in the usage data. |
| **3. Contextual data. Data indirectly related to the running of the intervention which may be influential over usage and analysis.** | |
| **3.1. External factors. Structures and events which may influence participation in the intervention.** | |
| How are users recruited to the intervention? | Either referred to programme by a healthcare professional, or have referred themselves via a self-completion online questionnaire. |
| Did any specific large-scale events, with the potential to impact on the intervention, occur during the period of the intervention (e.g. changes in treatment, health campaigns, illness outbreak, technical issues with the intervention)? | Covid-19.  Usage data cohort is users registered during a second national lockdown. Also note Christmas during and New Year during this time (e.g. new year’s resolutions, etc.) for providers C and D. |
| **3.2. Previous theory and findings. Results of behavioural analyses carried out during intervention development (e.g. logic models), and analyses of clinical outcomes if available.** | |
| What are the hypothesized mechanisms of the intervention (e.g. as specified in the intervention’s logic model)? | No specific logic model. Intervention based on a number of different theories and constructs, some including self-determination theory social cognitive theory and COM-B model. Researcher-developed logic model specifies information giving, followed by intention to change behaviour, followed by a self-regulatory cycle of behaviour change (including goal setting, self-monitoring, problem solving, feedback). |
| Which factors are identified as important in qualitative research, and can they be related to the variables collected in the trial (e.g. preferences for specific pages)? | N/A. |
| Which variables are identified as relating to outcomes (e.g. behavioural determinants, theoretical constructs, health factors)? | Unaware of whether this has been done. |

**Stage 2 checklist for the Analysing and Measuring Usage and Engagement Data (AMUsED) framework**

| **Selecting usage variables and generating research questions** | |
| --- | --- |
| **Generic questions by data type** | |
| **1. Descriptions of usage variables. Which usage variables are relevant to the intervention and in which format (e.g. number of users/sessions, duration, percentage of total, dichotomous)?** | |
| Completing intervention/trial period (stage1; 1.1 & 2.2). E.g. How many users complete the trial? What is the average time taken to complete? | - Number of times users set a goal.  - Number of times users self-monitor a behaviour/outcome.  - Number of unique articles accessed (providers A and D).  - Number of articles sent to users (provider C).  - Number of posts made, comments made and likes on posts in group forum (providers C, D).  - Number of calls with health coach (providers A and C).  - Number of minutes spent on the app (providers A, C and D).  The above are collected any time an instance occurs, and data can be broken down into engagement periods (30-day periods) across the 9-months. |
| Logins or sessions where the intervention was accessed (stage 1; 1.1 & 2.2). E.g. How many users start/complete each login/session? How long does it take to complete each session? How many pages are viewed within the session? Which session has the highest proportion of pages viewed, or duration of time spent on it? | Purpose of login: to track behaviours and outcomes, set new goals, interact with group forum or to engage with messages sent from health coach. See above. |
| Date of login and usage. E.g. When do users login? What time of year? Are there changes in frequency of logins? | Does engagement differ between engagement periods? E.g. more or less engagement earlier or later in the programme?  Could compare variables described above (goals, forum, messages) across engagement periods. |
| Time of day of login and usage. E.g. What time of day is usage? Are users more likely to spend longer on the intervention at certain times? | N/A. |
| Days/weeks of usage (stage1; 1.1 & 2.2). E.g. For how many days/weeks out of the total is the intervention accessed for? How many times within a week is the intervention accessed? Are there repeated uses within the same day? | Intervention is accessed over 9-month period, users do not have to access at specific times.  Providers A and D – there are structured education components to the programme, articles get ‘unlocked’, but users do not have to access at particular times.  There can be repeated uses within the same day, e.g. could fill in a food diary after each meal, could log PA at multiple time-points, could access different articles, videos or group forum at any time.  Providers A and D have sent aggregated data, broken down per engagement period.  Provider C has sent individual usage data (i.e. an entry for every engagement with the app). |
| Response to prompts/notifications (e.g. requests to login, email, text, upload data) (stage1; 1.1 & 2.2). E.g. How many responses are sent? How long after receiving notification do users take to log-in or respond? | N/A. |
| Features/linked menu components used (stage 1; 1.1, 1.2 & 2.2). E.g. How many features/components are accessed? How many users access each one? Which are completed and by how many users? Which feature/component has the highest proportion of pages viewed or time spent? What order are they viewed in? Is this the anticipated order? Which have the highest proportion of drop-out? | - Number of times users set a goal.  - Number of times users self-monitor a behaviour/outcome.  - Number of unique articles accessed (providers A and D).  - Number of articles sent to users (provider C).  - Number of posts made, comments made and likes on posts in group forum (providers C, D).  - Number of calls with health coach (provider C).  - Number of minutes spent on the app (providers A, C and D).  The above are collected any time an instance occurs, and data can be broken down into engagement periods (30-day periods) across the 9-months. |
| Revisiting components/features (stage 1; 1.1, 1.2 & 2.2). E.g. Are any used repeatedly? How many times are they revisited, and for how long? Which are most revisited? | There is data on the number of instances of monitoring/goals set/articles/forum. |
| Type of content/BCTs used (excluding administration pages) (stage 1; 1.2 & 2.2). E.g. How many groups of pages with similar content are accessed and by how many users? How many pages within the group are used? How many users view each page? Which groups of pages have the highest proportion of views? Which pages are viewed at each login, and when is the largest amount of pages viewed? Which pages have higher drop-out? | See features/menu components.  Could look at:  - How many users set and monitor behavioural goals (could compare by type?) and outcome goals during each engagement period **(goal setting and self-monitoring)**;  - How many users engage with the forum and how they engage, i.e. comment, like, post, etc. during each engagement period **(social support)**;  - What BCTs users are exposed to via educational materials sent via health coach messages (provider C) |
| Completing ongoing measures (e.g. monthly questionnaires, response options within content pages, uploading information or text responses) (stage 1; 2.1 & 2.2). E.g. How many users complete ongoing measures? When do they complete them? Do they also access the intervention at that time? | Number of users continuing to monitor progress against their behavioural and outcome goals (and set new ones) across engagement periods.  Number of users tracking their behaviours and weight across engagement periods.  Number of users continuing engagement with group forums across engagement periods.  Number of users accessing educational content across engagement periods. |
| External device usage (e.g. wearables and other sensor technologies) (stage 1; 2.3). E.g. How much time is spent with the device? How many times is it used? What number of days/weeks is it used for? | Number of users who monitor their steps?  Number of users who record activity though a wearable device? |
| **2. Relationships between usage and participant characteristics. Are users’ demographic, physical or psychosocial characteristics at baseline related to intervention usage?** | |
| Are any characteristics at baseline related to usage? E.g. Is anxiety associated with revisiting features? Is current health related to usage of external devices? Are users who spend more time on the intervention older than those who spend less time? Which characteristics are associated with drop-out? | N/A. |
| Are any contextual factors associated with usage (stage1; 3)? E.g. Is manner of recruitment related to usage? | N/A. |
| Do high/low users differ by other usage factors? E.g. Do users who spend more time on the intervention view more types of content than users who spend less time? Is usage of an external device related to intervention usage? | N/A. |
| **3. Relationships between usage, behavioural determinants, and target behaviours. Which usage variables are associated with follow-up measures for target behaviour and behavioural determinants? Which usage variables help explain changes in behaviour across the intervention?** | |
| Are baseline measures for behavioural determinants/target behaviour related to usage? E.g. Is the number of days the intervention is used for related to a behavioural determinant? Do users with low target behaviours spend less time on the intervention? | N/A – no baseline measures taken. |
| Which usage variables are related to behavioural determinants/target behaviours and at follow-up? E.g. Do users who view a group of pages containing a specific BCT score higher/lower for the associated behavioural determinant? Is completing/not completing a particular component associated with target behaviour at follow-up? Is the time spent on a session related to target behaviour? | Unlikely to be possible due to low numbers – is the cumulative time spent on the app per engagement period linked to a reduction in self-reported weight? |
| Is usage associated with measures for acceptability/satisfaction at follow-up? E.g. Are high levels of satisfaction associated with accessing more pages? Do users with low satisfaction spend less time using external devices? | N/A. |
| Do users who report positive changes in behavioural determinants/target behaviour from baseline to follow-up use the intervention differently to those who do not? E.g. Do users who report positive increases in a behavioural determinant view more pages from a specific component containing an associated BCT? Do users who report positive behaviour change spend more time on the intervention? | N/A. |
| Are relationships between usage and target behaviour moderated by demographic, psychosocial or health factors? E.g. Is the relationship between time spent on the intervention and target behaviour altered when moderated by anxiety? | N/A. |
| What level of usage is necessary for ‘effective engagement’? E.g. Do outcome measures plateau after viewing certain content, or after a certain amount of time or sessions completed? | Unlikely to be possible due to low numbers and not everyone setting a weight goal – do self-reported weights plateau after specific engagement periods? (E.g. is there a plateau in weight during later engagement periods?) |

**Stage 3 checklist for the Analysing and Measuring Usage and Engagement Data (AMUsED) framework**

| **Preparation for analysis** | |
| --- | --- |
| **Generic questions by data type** | |
| **1. Resources** | |
| What is the timeframe for completing the analyses? | Analysis completed by June 2022, to allow write up results for paper submission and final NIHR report in September 2022. |
| What resources are needed? E.g. additional research time, expertise | Expertise required to help with analysis plan, e.g. colleagues in Bath, and UoM colleagues working on HEDLINE project. |
| Is a plan of analysis already available? How does the analysis plan developed using the framework compare to that plan? Are changes or updates needed? | No other plan is available. |
| Is ethical clearance in place to carry out usage analyses? | Yes, and relevant NDA’s and DPA’s in place with providers who requested it. |
| **2. Selecting types of analysis and analytical software** | |
| Will the usage data be triangulated with qualitative data? | No, but we do have qualitative data (separate publication) to help interpret the results. |
| What analytical tools are available? | Excel and SPSS. |
| Is there sufficient statistical power to answer the planned research questions? | No, analyses will be exploratory. |
| Can the selected measures of usage be analysed using the available tools? Is bespoke software necessary (e.g. visualisation techniques)? | Yes, but advice and support required to choose the most appropriate statistical package for analysis. |
| **3. Data preparation** | |
| When is the data available? | Providers sending data between April 2021 and May 2022. |
| Is the data raw or has it been used/cleaned previously? | Raw log-data from provider C. Providers A and D have aggregated data. |
| How many datasheets are there? Will these need to be amalgamated? | Numerous data sheets from each provider. These will need to be amalgamated per provider and then cleaned data from the four providers will be combined for analysis. |
| Is the data structured to work with the tools available? What formats are the datasheets in (e.g. excel, .csv) and will they need converting for analysis? | Data sheets are Excel. Data will need to be uploaded to SPSS after data cleaning. |
| What preparation does the data need (e.g. cleaning, anonymizing)? | Data needs cleaning and combining. |
| Are all variables readily available or will they need extracting/transforming/recoding? | Data will need extracting and recoding. Transform data to one row per user per day for each engagement period for provider C, already aggregated for providers A and D. |
| Is the data in the right format to answer the research questions? Will it need adapting (e.g. continuous variables changed to categorical)? | Data is in the right format. |

**Appendix 2: Usage Data Field Definitions**

| **Data field** | **Definition** |
| --- | --- |
| **Self-monitoring** | |
| Number of times behaviours were self-monitored | Number of times a service user monitors/tracks a behaviour in the app during an engagement period. If users self-monitored more than one individual behaviour during one occasion, this was reported as individual instances of self-monitoring behaviours.  [Provider A can track diet, physical activity, fluid intake, mood, appetite, bowel movements, symptoms].  [Provider C can track exercise, steps, diet, sleep, alcohol, smoking, medicine, pain, mood, life rules (unspecified behaviour)].  [Provider D can track food, steps, sleep]. |
| Number of times outcomes were self-monitored | Number of times a service user monitors/tracks an outcome in the app during an engagement period. If users self-monitored more than one individual outcome during one occasion, this was reported as individual instances of self-monitoring outcomes.  [Provider A can track weight, blood glucose levels, blood pressure, weight circumference].  [Provider C can track weight, waist-hip ratio, fasting blood sugar, blood sugar, blood pressure].  [Provider D can track weight]. |
| **Goal setting** | |
| Number of times a behavioural and/or outcome goal was set or amended | Number of times a behavioural and/or outcome goal has been set or amended in the app during an engagement period. If users had set a goal for more than one individual behaviour/outcome during one occasion, this was reported as individual instances of goal setting.  [Provider A – goals relate to diet, physical activity, fluid intake, mood, appetite, bowel movements, symptoms, weight, blood glucose levels, blood pressure, weight circumference].  [Provider C – goals relate to exercise, steps, diet, sleep, alcohol, smoking, medicine, pain, mood, life rules (unspecified behaviour), weight, waist-hip ratio, fasting blood sugar, blood sugar, blood pressure].  [Provider D – goals relate to habits that have been prompted in educational material, e.g. physical activity, diet, mind-set, stress, sleep, custom (free-text), weight]. |
| Number of times a behavioural goal was set | [Only applicable to Providers C and D].  If users had set a goal for more than one individual behaviour during one occasion, this was reported as individual instances of setting behavioural goals.  Number of times a behavioural goal has been set in the app during an engagement period.  [Provider C – goals relate to exercise, steps, diet, sleep, alcohol, smoking, medicine, pain, mood, life rules (unspecified behaviour)].  [Provider D – goals relate to diet, physical activity, mind-set, stress, sleep, custom (free-text)]. |
| Number of times an outcome goal was set | [Only applicable to Providers C and D].  If users had set a goal for more than one individual outcome during one occasion, this was reported as individual instances of setting outcome goals.  Number of times an outcome goal has been set in the app during an engagement period.  [Provider C – goals relate to weight, waist-hip ratio, fasting blood sugar, blood sugar, blood pressure].  [Provider D – goals relate to weight]. |
| **Educational content** | |
| Number of times any articles were accessed | [Only applicable to providers A and D]. As this is a measure of 'the number of times' an article is accessed in the app, this could include instances where the service user accesses an 'article' more than once within the engagement period. |
| Number of unique articles that were accessed | [Only applicable to providers A and D].  As this is a measure of 'the number of unique articles' accessed in the app, this only includes the first instance that a service user accesses an 'article' within the engagement period. |
| Number of times educational content was sent from health coach | [Only applicable to provider C].  The number of times educational content was sent from the health coach to the service user in the app during an engagement period. Educational content might include PDF articles, videos, or links to external websites (e.g. links to recipes, exercise videos, support). |
| **Health coach support** | |
| Number of phone/video calls with health coach | Number of phone/video call conversations in the engagement period that took place between the coach and the service user  [Provider D does not offer phone calls as part of their service provision]. |
| Number of messages to health coach that have been responded to | [Only applicable to providers A and D].  Number of messages to the health coach by the service user in the app that have been responded to per engagement period. |
| Number of messages sent from health coach | [Only applicable to provider C].  The number of support messages sent from the health coach to the service user in the app during an engagement period. This includes any text messages, support videos or support links sent to the service user that did not otherwise contain educational content. |
| Number of messages sent from service user | [Only applicable to provider C].  The number of messages sent from the service user to the health coach during an engagement period. This includes any images (e.g. pictures of meals for coach feedback) and text messages sent to the health coach. |
| **Group support** | |
| Number of peer messages sent in group chat by service user | The definition of a message sent by the service user in a group chat during the engagement period. |
| Number of sent group posts in discussion forum by service user | Number of times service user posts on the group forum per engagement period.  [Provider A does not offer group forum support as part of their service provision]. |
| Number of sent group likes in discussion forum by service user | Number of times service user ‘likes’ another person’s post on the group forum per engagement period.  [Provider A does not offer group forum support as part of their service provision]. |
| Number of sent group comments in discussion forum by service user | Number of times service user comments on another person’s post in the group forum per engagement period.  [Provider A does not offer group forum support as part of their service provision]. |
| **App usage** | |
| Total time interaction on the app | Cumulative time SU spent in the app per engagement period. Note: if service users could complete the functions in the app I under 30 seconds, this would not be registered as app usage in the current datasets. |

**Appendix 3: Duration of engagement over the 9-month intervention**

Table A1. Total number of users who engaged with the apps during engagement periods 1, 5 and 9

|  | **Engagement period *^a^*** | | |
| --- | --- | --- | --- |
|  | **1** | **5** | **9** |
|  | ***n* (%)** | | |
| **No. of users who spent at least 1 minute on the app** | 1230 (67) | 1008 (55) | 667 (37) |
| **No of users who spent at least 5 minutes on the app** | 1139 (62) | 911 (50) | 564 (31) |
| **No of users who spent at least 10 minutes on the app** | 1076 (59) | 836 (46) | 502 (27) |

*Note.* n = 1,826 service users
*^a^* Engagement period denotes 30 day periods: engagement period 1 = days 1 to 30; engagement period 2 = days 31 to 60, etc.

**Appendix 4: Engagement with intervention features across the 9-month intervention, broken down by provider**

Table A2. Engagement with intervention features across the 9-month intervention, broken down by provider (median, IQR and range)

|  | **Engagement period *^a^*** | | | | | | | | |
| --- | --- | --- | --- | --- | --- | --- | --- | --- | --- |
|  | **1** | **2** | **3** | **4** | **5** | **6** | **7** | **8** | **9** |
|  | **Median (IQR) [Range]** | | | | | | | | |
| **Provider A *^b^*** | | | | | | | | | |
| **Self-monitoring** | | | | | | | | | |
| Number of times behaviours were self-monitored | 25 (77)  [0-659] | 9 (73)  [0-650] | 2 (61)  [0-680] | 0 (49)  [0-659] | 0 (34)  [0-682] | 0 (28)  [0-667] | 0 (12)  [0-661] | 0 (6)  [0-648] | 0 (6)  [0-662] |
| Number of times outcomes were self-monitored | 1 (3)  [0-59] | 0 (3)  [0-38] | 0 (3)  [0-66] | 0 (2)  [0-52] | 0 (0)  [0] | 0 (0)  [0] | 0 (0)  [0] | 0 (0)  [0] | 0 (0)  [0] |
| **Goal setting** | | | | | | | | | |
| Number of times a behavioural and/or outcome goal was set or amended | 17.5 (31)  [0-319] | 10 (41)  [0-300] | 5 (30)  [0-300] | 1 (30)  [0-300] | 0 (30)  [0-300] | 0 (24)  [0-300] | 0 (17)  [0-300] | 0 (12)  [0-256] | 0 (2)  [0-261] |
| **Educational content** | | | | | | | | | |
| Number of times articles were accessed | 7 (31)  [0-119] | 4 (25)  [0-125] | 2 (12)  [0-123] | 0 (8)  [0-150] | 0 (0)  [0-90] | 1 (4)  [0-143] | 2 (5)  [0-89] | 3 (5)  [0-197] | 3 (8)  [0-230] |
| Number of times unique articles were accessed | 7 (3)  [0-119] | 4 (25)  [0-125] | 2 (12)  [0-123] | 0 (8)  [0-150] | 0 (3)  [0-90] | 1 (4)  [0-143] | 2 (5)  [0-89] | 3 (5)  [0-197] | 2 (4)  [0-214] |
| **Health coach support** | | | | | | | | | |
| Number of calls with health coach | 0 (1)  [0-3] | 0 (1)  [0-4] | 0 (1)  [0-2] | 0 (1)  [0-2] | 0 (1)  [0-2] | 0 (1)  [0-3] | 0 (1)  [0-2] | 0 (1)  [0-2] | 0 (0)  [0-2] |
| Number of messages health coach has responded to | 0 (3)  [0-32] | 0 (5)  [0-40] | 0 (1)  [0-35] | 0 (0)  [0-20] | 0 (0)  [0-13] | 0 (0)  [0-9] | 0 (0)  [0-14] | 0 (0)  [0-27] | 0 (0)  [0-27] |
| **Provider C *^c^*** | | | | | | | | | |
| **Self-monitoring** | | | | | | | | | |
| Number of times behaviours were self-monitored | 59 (71)  [0-368] | 57 (80)  [0-356] | 51 (81)  [0-519] | 40 (98)  [0-433] | 32 (89)  [0-324] | 30 (86)  [0-360] | 19 (79)  [0-332] | 0 (63)  [0-354] | 0 (49)  [0-291] |
| Number of times outcomes were self-monitored | 2 (4)  [0-55] | 0 (3)  [0-35] | 0 (2)  [0-57] | 0 (1)  [0-56] | 0 (1)  [0-56] | 0 (2)  [0-30] | 0 (1)  [0-44] | 0 (1)  [0-29] | 0 (1)  [0-28] |
| **Goal setting** | | | | | | | | | |
| Number of times a behavioural and/or outcome goal was set or amended | 4 (3)  [0-23] | 0 (1)  [0-9] | 0 (0)  [0-13] | 0 (0)  [0-8] | 0 (0)  [0-8] | 0 (0)  [0-4] | 0 (0)  [0-3] | 0 (0)  [0-3] | 0 (0)  [0-5] |
| Number of times a behavioural goal was set | 3 (3)  [0-23] | 0 (1)  [0-9] | 0 (0)  [0-13] | 0 (0)  [0-8] | 0 (0)  [0-8] | 0 (0)  [0-3] | 0 (0)  [0-3] | 0 (0)  [0-3] | 0 (0)  [0-5] |
| Number of times an outcome goal was set | 1 (1)  [0-5] | 0 (0)  [0-3] | 0 (0)  [0-2] | 0 (0)  [0-2] | 0 (0)  [0-2] | 0 (0)  [0-1] | 0 (0)  [0-1] | 0 (0)  [0-1] | 0 (0)  [0] |
| **Educational content** | | | | | | | | | |
| Number of times educational content was sent from health coach | 8 (5)  [0-20] | 6 (4)  [0-13] | 6 (5)  [0-17] | 4 (4)  [0-10] | 4 (5)  [0-12] | 2 (4)  [0-14] | 1 (2)  [0-17] | 1 (2)  [0-10] | 0 (2)  [0-18] |
| **Health coach support** | | | | | | | | | |
| Number of calls with health coach | 1 (0)  [0-2] | 0 (0)  [0-1] | 0 (0)  [0] | 0 (0)  [0-1] | 0 (1)  [0-1] | 0 (0)  [0-2] | 0 (0)  [0-1] | 0 (0)  [0] | 0 (0)  [0-1] |
| Number of messages sent from health coach | 5 (1)  [0-11] | 4 (1)  [0-9] | 4 (2)  [0-14] | 2 (3)  [0-10] | 2 (1)  [0-6] | 2 (3)  [0-7] | 1 (2)  [0-5] | 1 (1)  [0-4] | 1 (1)  [0-4] |
| Number of messages sent from service user | 4 (6)  [0-191] | 2 (4)  [0-135] | 2 (5)  [0-102] | 1 (3)  [0-146] | 1 (2)  [0-131] | 0 (2)  [0-46] | 0 (1)  [0-35] | 0 (1)  [0-30] | 0 (1)  [0-30] |
| **Group support** | | | | | | | | | |
| Number of group posts in discussion forum by service user | 0 (0)  [0-12] | 0 (0)  [0-2] | 0 (0)  [0-1] | 0 (0)  [0-1] | 0 (0)  [0] | 0 (0)  [0] | 0 (0)  [0] | 0 (0)  [0-1] | 0 (0)  [0-1] |
| Number of comments on group posts in discussion forum by service user | 0 (0)  [0-13] | 0 (0)  [0-12] | 0 (0)  [0-6] | 0 (0)  [0-2] | 0 (0)  [0-1] | 0 (0)  [0] | 0 (0)  [0] | 0 (0)  [0] | 0 (0)  [0-2] |
| Number of likes on group posts in discussion forum by service user | 0 (42)  [0-42] | 0 (0)  [0-40] | 0 (0)  [0-9] | 0 (0)  [0-6] | 0 (0)  [0-6] | 0 (0)  [0-3] | 0 ()  [0-2] | 0 (0)  [0-1] | 0 (0)  [0-1] |
| **Provider D *^d^*** | | | | | | | | | |
| **Self-monitoring** | | | | | | | | | |
| Number of times behaviours were self-monitored | 33 (106)  [0-3214] | 30 (115)  [0-1483] | 7 (68)  [0-616] | 1 (35)  [0-507] | 0 (18)  [0-341] | 0 (8)  [0-320] | 0 (2)  [0-374] | 0 (0)  [0-288] | 0 (0)  [0-292] |
| Number of times outcomes were self-monitored | 6 (12)  [0-85] | 5 (10)  [0-147] | 3 (8)  [0-116] | 1 (6)  [0-98] | 0 (4)  [0-138] | 0 (3)  [0-102] | 0 (2)  [0-64] | 0 (0)  [0-241] | 0 (0)  [0-113] |
| **Goal setting** | | | | | | | | | |
| Number of times a behavioural and/or outcome goal was set or amended | 2 (3)  [0-27] | 0 (1)  [0-13] | 0 (0)  [0-12] | 0 (0)  [0-9] | 0 (0)  [0-6] | 0 (0)  [0-8] | 0 (0)  [0-14] | 0 (0)  [0-12] | 0 (0)  [0-4] |
| Number of times a behavioural goal was set | 1 (3)  [0-27] | 0 (1)  [0-12] | 0 (0)  [0-10] | 0 (0)  [0-8] | 0 (0)  [0-6] | 0 (0)  [0-7] | 0 (0)  [0-5] | 0 (0)  [0-10] | 0 (0)  [0-3] |
| Number of times an outcome goal was set | 0 (0)  [0-10] | 0 (0)  [0-4] | 0 (0)  [0-3] | 0 (0)  [0-4] | 0 (0)  [0-4] | 0 (0)  [0-2] | 0 (0)  [0-9] | 0 (0)  [0-4] | 0 (0)  [0-2] |
| **Educational content** | | | | | | | | | |
| Number of times articles were accessed | 28 (87)  [0-846] | 12 (69)  [0-636] | 2 (54)  [0-400] | 0 (9)  [0-335] | 0 (0)  [0-385] | 0 (0)  [0-316] | 0 (0)  [0-201] | 0 (0)  [0-182] | 0 (0)  [0-249] |
| Number of times unique articles were accessed | 6 (19)  [0-40] | 2 (21)  [0-42] | 0 (17)  [0-65] | 0 (1)  [0-25] | 0 (0)  [0-12] | 0 (0)  [0-4] | 0 (0)  [0-1] | 0 (0)  [0-2] | 0 (0)  [0-1] |
| **Health coach support** | | | | | | | | | |
| Number of messages health coach has responded to | 6 (18)  [0-185] | 4 (16)  [0-323] | 1 (9)  [0-339] | 0 (4)  [0-859] | 0 (1)  [0-802] | 0 (0)  [0-280] | 0 (0)  [0-302] | 0 (0)  [0-518] | 0 (0)  [0-513] |
| **Group support** | | | | | | | | | |
| Number of peer messages sent in group chat by service user | 2 (11)  [0-152] | 1 (9)  [0-246] | 0 (5)  [0-285] | 0 (2)  [0-470] | 0 (0)  [0-259] | 0 (0)  [0-68] | 0 (0)  [0-40] | 0 (0)  [0-22] | 0 (0)  [0-31] |
| Number of group posts in discussion forum by service user | 0 (0)  [0] | 0 (0)  [0-1] | 0 (0)  [0-1] | 0 (0)  [0-6] | 0 (0)  [0-10] | 0 (0)  [0-13] | 0 (0)  [0-4] | 0 (0)  [0-6] | 0 (0)  [0-9] |
| Number of comments on group posts in discussion forum by service user | 0 (0)  [0] | 0 (0)  [0-30] | 0 (0)  [0-75] | 0 (0)  [0-220] | 0 (0)  [0-189] | 0 (0)  [0-58] | 0 (0)  [0-175] | 0 (0)  [0-278] | 0 (0)  [0-274] |
| Number of likes on group posts in discussion forum by service user | 0 (0)  [0-48] | 0 (0)  [0-49] | 0 (0)  [0-177] | 0 (0)  [0-64] | 0 (0)  [0-79] | 0 (0)  [0-107] | 0 (0)  [0-98] | 0 (0)  [0-173] | 0 (0)  [0-196] |

*Note.* Provider B was unable to supply usage data for this analysis. Providers are labelled A, C and D to provide anonymisation, but to allow cross-reference to previous papers published by the research team for this programme of work.
*Note.* The numbers in curved brackets denote the interquartile range (IQR). The numbers in squared brackets denote the range.
*Note.* If users self-monitored more than one individual behaviour/outcome during one occasion, or set a goal for more than one individual behaviour/outcome during one occasion, this was reported as individual instances of self-monitoring or goal setting.
*^a^* Engagement period denotes 30 day periods: engagement period 1 = days 1 to 30; engagement period 2 = days 31 to 60, etc.
*^b^* n = 940 service users.
*^c^* n = 283 service users.
*^d^* n = 603 service users.
